# Amniotic Fluid Stem Cells Inhibit the Progression of Bleomycin-Induced Pulmonary Fibrosis via CCL2 Modulation in Bronchoalveolar Lavage

**DOI:** 10.1371/journal.pone.0071679

**Published:** 2013-08-13

**Authors:** Orquidea Garcia, Gianni Carraro, Gianluca Turcatel, Marisa Hall, Sargis Sedrakyan, Tyler Roche, Sue Buckley, Barbara Driscoll, Laura Perin, David Warburton

**Affiliations:** 1 Developmental Biology and Regenerative Medicine Program, Saban Research Institute, Children’s Hospital Los Angeles, Los Angeles, California, United States of America; 2 Integrative Biology of Disease Graduate Program, Keck School of Medicine, University of Southern California, Los Angeles, California, United States of America; 3 Division of Urology, Saban Research Institute, Children’s Hospital Los Angeles, Los Angeles, California, United States of America; University of Pittsburgh, United States of America

## Abstract

The potential for amniotic fluid stem cell (AFSC) treatment to inhibit the progression of fibrotic lung injury has not been described. We have previously demonstrated that AFSC can attenuate both acute and chronic-fibrotic kidney injury through modification of the cytokine environment. Fibrotic lung injury, such as in Idiopathic Pulmonary Fibrosis (IPF), is mediated through pro-fibrotic and pro-inflammatory cytokine activity. Thus, we hypothesized that AFSC treatment might inhibit the progression of bleomycin-induced pulmonary fibrosis through cytokine modulation. In particular, we aimed to investigate the effect of AFSC treatment on the modulation of the pro-fibrotic cytokine CCL2, which is increased in human IPF patients and is correlated with poor prognoses, advanced disease states and worse fibrotic outcomes. The impacts of intravenous murine AFSC given at acute (day 0) or chronic (day 14) intervention time-points after bleomycin injury were analyzed at either day 3 or day 28 post-injury. Murine AFSC treatment at either day 0 or day 14 post-bleomycin injury significantly inhibited collagen deposition and preserved pulmonary function. CCL2 expression increased in bleomycin-injured bronchoalveolar lavage (BAL), but significantly decreased following AFSC treatment at either day 0 or at day 14. AFSC were observed to localize within fibrotic lesions in the lung, showing preferential targeting of AFSC to the area of fibrosis. We also observed that MMP-2 was transiently increased in BAL following AFSC treatment. Increased MMP-2 activity was further associated with cleavage of CCL2, rendering it a putative antagonist for CCL2/CCR2 signaling, which we surmise is a potential mechanism for CCL2 reduction in BAL following AFSC treatment. Based on this data, we concluded that AFSC have the potential to inhibit the development or progression of fibrosis in a bleomycin injury model during both acute and chronic remodeling events.

## Introduction

IPF is a chronic, progressive and fatal lung disease, surmised to result from a myriad of factors. The improvement of diagnostic technology and criteria, coupled with an increase in aged populations worldwide, virtually ensures that morbidity and mortality attributed to IPF will increase [1]. The histopathology of IPF demonstrates a characteristic heterogeneity: areas of normal parenchyma interspersed with areas of paraseptal and subpleural fibrosis [2]. At the cellular level IPF is characterized by alveolar epithelial injury, the initiation of inflammatory cascades, exaggerated pro-fibrotic cytokine expression, increased extracellular matrix deposition, and the development of fibrotic lesions termed ‘foci’ [2]–[5]. The only effective and definitive treatment for IPF is lung transplantation; however this option is limited by the quality and availability of donor lungs.

Recently, treatment strategies for IPF have focused on immunomodulation of cytokine “biomarker” targets [6]. In particular, expression of the pro-fibrotic cytokine CCL2 plays a significant role in IPF as previous studies indicate that it is primarily secreted by type II alveolar epithelia (AECII) and its secretion is significantly increased during inflammatory and fibrotic remodeling events in the lung [7]–[11]. Furthermore, in experimental models of lung fibrosis, increased expression of CCL2 attracts fibroblasts, and stimulates their collagen secretion and proliferation [12]–[14]. Inhibition of CCL2 production, deletion of CCR2 (the high affinity receptor for CCL2), or CCL2/CCR2 antagonism, inhibits the deposition of collagen and attenuates the experimental development of fibrosis [15]–[19]. Thus emerges the importance of CCL2/CCR2 signaling in the pathogenesis of pulmonary fibrosis.

As traditional pharmacological therapies for treatment of IPF have failed to yield FDA approved treatments, the use of exogenous stem and progenitor cells to ameliorate injury and induce endogenous repair mechanisms within the context of IPF has become a therapeutic target in recent years [20]–[23]. The potential ability of cell based therapies to inhibit experimentally induced pulmonary fibrosis, while conferring multiple simultaneous beneficial effects, have further peaked interest in this treatment strategy [24]–[26]. Various groups have described anti-fibrotic actions of multiple cell populations derived from organ specific or hematopoetic, mesenchymal, embryonic and extra-embryonic origins [25], [27]–[34]. While the merits of the efficacy of cell populations derived from various origins remains highly debated, the particular use of MSC for the treatment of fibrotic disease remains controversial in the literature as their pro-fibrotic versus anti-fibrotic mechanisms of action are still debated [35]–[37]. Recent investigations into the paracrine mechanisms of stem and progenitor populations have indicated that secreted factors derived from these cell based therapies may in fact be the key to their anti-fibrotic properties, making them potentially more effective than single agent therapeutic strategies [38], [39].

We have previously investigated the therapeutic potential of a distinct population of multipotent cells isolated from amniotic fluid based on the expression of c-kit known as Amniotic Fluid Stem Cells (AFSC) [40]. AFSC express stem cell markers found in Embryonic Stem Cells (ESCs) such as Oct4 and SSEA-4 and can be induced to differentiate into cell lineages of all three embryonic germ layers without forming terratomas *in vivo* [41]. AFSC are negative for hematopoetic markers: CD34, CD45 and CD133, but do express CD29, CD44, CD73 and CD105, markers which are also found on mesenchymal and neuronal stem cells [42]. Recent studies have demonstrated the potential for AFSC reprogramming into a pluripotent state through simple manipulation of culture media [43]. In both acute and chronic-fibrotic injury models, our studies have demonstrated the immunomodulatory functions of AFSC in the kidney, which correlate with decreases in pro-fibrotic cytokines such as CCL2, improvements in organ function, inhibition of the development of interstitial fibrosis and increased life span in experimental animals [44], [45].

In the present study, we used the murine bleomycin injury model to induce the parenchymal remodeling, increased collagen expression and elevated CCL2 production seen in human IPF [46]–[49]. We then treated cohorts intravenously with murine AFSC to test whether AFSC can inhibit the progression of experimentally induced pulmonary fibrosis [47], [48]. We determined that AFSC treatment, administered during what we termed “acute” or “chronic” fibrotic remodeling events, inhibited changes in histology, collagen deposition and pulmonary function associated with the development of pulmonary fibrosis. We also observed that AFSC express CCR2, the high affinity receptor for CCL2, appear to home to fibrotic foci *in vivo* and inhibit increased CCL2 levels in bronchoalveolar lavage (BAL) following bleomycin-induced lung injury. Through *in vitro* migration assays, we discovered that AFSC do indeed migrate toward increased CCL2 concentrations found in bleomycin-injured BAL. Finally, we provide data in support of a potential mechanism for the reduction of CCL2 by AFSC: the proteolytic cleavage of CCL2 by AFSC secreted MMP-2, inducing formation of a previously described CCR2 receptor antagonist cleavage product [50]–[52].

The use of AFSC in a bleomycin injury model to inhibit the progression of fibrosis through the immunomodulation of pro-fibrotic cytokines demonstrates the use of a unique cell population that unlike mesenchymal stem cells (MSC), have not been hypothesized to contribute to development or exacerbation of fibrosis [35], [36]. Although the use of various cell populations to attenuate the progression of pulmonary fibrosis, with varying degrees of success, has been previously described, we are the first to demonstrate that AFSC directly respond to increased CCL2 gradients found in injured lung BAL. The observed retention of AFSC within fibrotic lesions, and their homing ability toward CCL2 gradients further suggests the potential for AFSC to deliver therapeutic effects specifically to sites of injury, which may provide another potential avenue in which AFSC therapy may prove to be superior to single agent non-specific therapies. Finally, we are the first to propose a potential mechanism for CCL2 reduction in BAL following AFSC treatment and to provide data in support of this hypothesized mechanism [25], [27], [28], [33]. This novel cell based therapy and proposed mechanism thus suggests the translational potential for AFSC to arrest the progression of pulmonary fibrosis at the stage at which AFSC are administered.

## Methods

### Ethics Statement

Samples of human amniotic fluid from male fetuses (12–18 weeks of gestation) were provided to our laboratory by Genzyme Genetics Corporation (Monrovia, CA, USA) after karyotyping analysis. No written or verbal consent was required since samples were not identified and information obtained about the samples was limited to karyotype and fetal health status. All animal studies were performed in adherence to the National Institutes of Health Guide for the Care and Use of Laboratory Animals and approved by and performed according to the protocols and guidelines of the Institutional Animal Care and Use Committee at Children’s Hospital Los Angeles (Animal Welfare Assurance Number: A3276-01). All surgery was performed under isoflurane anesthesia, and every effort was made to minimize suffering.

### Isolation and Culture of AFSC

The isolation, culture and characterization of the pluripotency of human and mouse AFSC is a well established protocol in our laboratory, and clones used in these experiments are the same as those used in our previous publications [42], [44], [45], [53], [54]. Samples of murine amniotic fluid, used in all *in vivo* experiments, were obtained from E13.5 embryos and samples of human amniotic fluid, used in *in vitro* experiments, were obtained from amniocentesis samples form 12–18 weeks of gestation. Briefly, the stem cell population was isolated from the general amniotic cellular milieu using standard Magnetic Sorting (MACS) techniques (Miltenyi Biotech, Auburn, CA) against the cell surface marker, c-kit. Clones derived from a single sample of amniotic fluid were cultured in petri dishes in medium containing α-MEM Medium, 20% Fetal Bovine Serum, 1% L-Glutamine and 1% antibiotics (pen-strep) (Gibco/BRL, Rockville, MD) supplemented with 20% Chang Medium B and 2% Chang Medium C (Irvine Scientific, Santa Ana, CA). All *in vivo* AFSC treatment experiments were conducted with murine AFSC obtained from embryos of the same background as the experimental animals. Prior to injection, a clonal murine AFSC population was labeled with a cell surface marker, chloromethylbenzamine-1,1′-diactaolecyl-3,3,3′,3′-tetramethylindocarbocyanine perchlorate (CM-Dil) (Invitrogen, Carlsbad, CA) according to the manufacturers specifications, in order to track the cells after injection.

### Bleomycin Induced Lung Injury and AFSC Treatment

Female C57Bl/6J mice 8–12 wks. of age (Jackson Laboratories, Bar Harbor, Maine) were randomly selected, for bleomycin-injury or saline controls; a minimum of 6 mice were used for each experimental condition, experimental analysis and time point. For bleomycin treatment, 1.5 U/kg bleomycin (Sigma, St. Louis, MO) was dissolved in 50 µl of saline and injected into the trachea using a sterile 28_G_ needle under isoflurane anesthesia. Mice were housed in plastic cages on a 12-hour light/12-hour dark cycle with access to food and water ad libitum until harvest. Bleomycin injured cohorts not receiving murine AFSC treatments were sacrificed at 14 or 28 days post bleomycin-injury. AFSC treated mice received 1×10^6^ murine AFSC intravenously (IV) in a 50 µl volume of sterile PBS at either 2 hours (Bleo+AFSC day 0) or 14 days (Bleo+AFSC day 14) post-bleomycin-injury. Mice receiving murine AFSC on day 0 were sacrificed at either 3 days (acute time point) or 28 days (chronic time point) post-bleomycin. Mice receiving murine AFSC on day 14 were sacrificed at 28 days post-bleomycin.

### Histology

Whole lung specimens, from a subset of animals randomly chosen from each experimental group, were fixed in 4% paraformaldehyde at 20–25 cm H_2_O inflation pressure, embedded in paraffin and cut into 5–7 µm thick sections. Mouse lung sections were stained with Sirius Red/Fast Green FCF (Sigma, St. Louis, MO) for collagen visualization. Morphological changes in 225 randomly chosen microscopic fields, spanning all experimental conditions at the chronic time point, were photographed with 20-fold magnification, and were quantified according to the numerical scale proposed by Ashcroft et al. [55]. Visualization and quantification of CM-Dil labeled cells was achieved through counterstaining with 4′,6-diamidino-2-phenylindole (DAPI) (Vector Laboratories, Burlingame, CA). *In vitro* adherent cells were fixed in 4% paraformaldehyde for staining. Overnight incubation with primary antibodies used for immunofluorescence included CCR2 [1 µg/ml] (abcam, Cambridge, MA) and α-smooth muscle actin (α-SMA) [4 µg/ml] (Sigma, St. Louis, MO).

### Measurements of Lung Mechanics and Collagen Quantification

A subset of animals randomly chosen from each experimental group were anesthetized with 70–90 mg/kg pentobarbital sodium solution, tracheotomized, placed in a plethysmograph and connected to the Scireq small animal ventilator (Scireq, Montreal, Canada) to measure pulmonary mechanics. Mice were mechanically ventilated at a rate of 150 breaths/min, tidal volume of 10 ml/kg, and positive end-expiratory pressure of 2–3 cmH2O. All maneuvers were computer controlled via Flexivent v5.2 software (Scireq, Montreal, Canada). Pressure-volume loops were generated by a sequential delivery of seven increments of air into the lungs from resting pressure to total lung capacity followed by seven expiratory steps during which air was incrementally released. Pressures at each of the incremental volumes delivered were recorded and graphed to give pressure-volume loops. The Salazar-Knowles equation was applied to measurements resulting from the pressure volume manipulations to calculate quasi-static compliance and hysteresis (the area enclosed by the pressure volume loop), which provides an estimate of the amount of airspace closure that existed before the P–V loop maneuver [56]. Negative pressure forced expirations were performed via rapidly switching the airway opening to negative pressure, resulting in the ability to measure Forced Vital Capacity (FVC). All measurements and maneuvers were performed in triplicate. Following pulmonary function testing, whole lungs were excised from each animal for total collagen content analysis via hydroxyproline assay kit (BioVision, Milpitas, CA) according to the manufacturers instructions. Briefly, whole lungs were washed in PBS, weighed and homogenized in 100 µl dH_2_O/mg of tissue. Tissue samples were then hydrolyzed at 120°C for 3 hours before transfer to a 96 well plate for oxidation of free hydroxyproline. Hydroxyproline content was then assessed by spectrophotometry at 570 nm.

### Collection of Bronchoalveolar Lavage (BAL) and Lung Tissue

Randomly chosen cohorts from each experimental condition and time point were sacrificed using 100 mg/kg intraperitoneal injection of pentobarbital sodium solution. The chest cavity was then opened for tissue retrieval creating a pneumothorax. Subjects were exsanguinated via injection of 0.9% saline into the right ventricle. BAL was collected through three separate washes consisting of the addition of 0.5 cc phosphate buffered saline into the trachea via a 3 cc syringe attached to a 20_G_ angiocatheter followed by gentile aspiration. BAL was spun at 1500×g for 5 minutes at 25°C to separate the cellular components. For standardization purposes during experimental analyses, total volume of BAL recovered following lavage was recorded, and volume measurements were used in standardization calculations for ELISA and MMP-2 activity assays: BAL volume recovered (ml) was multiplied by (concentration/ml) to give concentration/lung. In addition, determination of total protein concentration in BAL supernatants was determined via the Bradford method using an assay kit (Bio-Rad Laboratories, Hercules, CA) for zymography and Western blotting. BAL supernatants were frozen at −20°C, and cellular fractions were cytospun and stained with the Diff-Quick Stain Kit (IMEB Inc., San Marcos, CA) for differential analysis. Following BAL collection, the entire lung of each animal was harvested for AECII according to our previously published protocol [57], or removed from the chest cavity for whole lung homogenization. AECII were isolated from lavaged lungs by dispase digestion followed by differential adherence on IgG plates. Homogenized lungs were, washed briefly in PBS and homogenized in 100 µl PBS/mg of tissue with protease inhibitors before the addition of Triton X-100 to 1%. Homogenates were vortexed, and both AECII and lung homogenates were kept at −80°C until use. Prior to use, the total protein in all AECII and lung homogenate samples was quantified using the Bradford assay kit previously described for use in standardization of experimental analyses.

### Proteomic Cytokine Analysis

Cytokine levels were assessed in BAL and homogenized lung tissue samples using an equal amount of protein (300 µg) incubated with multiplex cytokine assay membranes (R&D Systems, Minneapolis, MN). Membranes were developed with Super Signal West Pico Chemiluminescent Substrate (Thermo Fisher Scientific Inc., Rockford, IL), scanned and analyzed using Image J Software.

### CCL2 ELISA

BAL samples were analyzed for CCL2 concentration, and standardized against total volume recovered, using the mouse MCP-1 ELISA KIT (Invitrogen, Camarillo, CA) according to the manufacturer’s suggested protocol with spectrophotometry at 450 nm.

### In Vitro Collagen Assay

To study the ability of the damaged alveolar cytokine milieu, as reflected by BAL, specifically CCL2, to induce collagen synthesis in fibroblasts we developed an *in vitro* assay system to measure newly synthesized soluble collagen. Murine 3T3 fibroblasts (ATCC, Manassas, VA) were plated at 4×10^4^ cells per well in 24 well plates and cultured in DMEM +10% FBS +1% penicillin-streptomycin. Once confluent, duplicates of 100 µl of BAL (n = 6 per time point), conditioned media (n = 10) or recombinant murine CCL2 at 0, 50, 100 or 200 pg/ml (R&D Systems, Minneapolis, MN), was added to each culture for an additional 48 hours. At the end of the 48-hour growth period, conditioned media supernatants were removed; adherent cells were washed in PBS, and incubated with 0.5 M acetic acid for 2 hours. Acetic acid fractions containing newly synthesized solubilized pro-collagen were then collected and assayed according to the manufacturers instructions via the Sircol Assay system (Biocolor Life Sciences, County Antrim, UK) and measured with spectrophotometry at 570 nm.

### Western Blotting and Zymography

BAL samples were concentrated using Amicon Ultra-4 Centrifugal Filter Units (3 kDa) (Milipore, Bilerica, MA). To determine the slight shift in molecular weight due to cleavage of the CCL2 ligand, we performed Western blotting using gradient gels with high resolving capacity and reagents specific for detection of low molecular weight proteins. 20 µg total protein from BAL or cell lysate was loaded onto 4–12% Bis-Tris Gels with MOPS buffer (Novex, Grand Island, NY). Anti-CCL2 [1∶200] (R&D Systems, Minneapolis, MN), Anti-Beta Actin [1∶1000] (Cell Signaling, Danvers, MA) or Anti-MMP-2 [1∶1000] (abcam, Cambridge, MA) antibodies were incubated with membranes overnight. BAL zymography was performed using either 10 or 20 µg protein (indicated in figure legend) on 10% gelatin gels (Novex, Grand Island, NY) with MMP-9 control (Amersham Life Science, Pittsburg, PA) and MMP-2 control collected from conditioned media from CCL-201 human lung fibroblasts (ATCC, Manassas, VA). Western blots and zymograms were each repeated a minimum of 3 times per sample.

### MMP-2 Activity Assay

BAL samples were analyzed for endogenous active MMP-2, using the mouse MMP-2 Activity Assay KIT (Quickzyme Biosciences, Netherlands) according to the manufacturer’s suggested protocol with spectrophotometry at 405 nm and standardized against total volume recovered.

### Migration Assay

Murine AFSC were assayed for migration toward recombinant mouse CCL2 (R&D Systems, Minneapolis, MN) at concentrations of 0, 25, 50, 75 and 100 pg/ml. Human AFSC were assayed for migration toward recombinant CCL2 at concentrations of 0, 12.5, 25 and 50 ng/ml. Murine AFSC migration toward duplicates of 100 µl BAL samples (n = 6 per time point, CCL2 concentrations having been previously determined via ELISA) from mice with acute and chronic bleomycin injury were also assayed. Furthermore, to determine the extent of acutely produced CCL2 on murine AFSC migration; a mouse CCL2 neutralization antibody (R&D Systems, Minneapolis, MN) was used on “acute” BAL samples in additional wells. Migration assays were performed according to the Boyden chamber method as previously described [53]. Media containing recombinant CCL2 or BAL was added to the well underneath the insert. As positive and negative controls DMEM and 0.1% BSA (random migration) or DMEM with 2.5% FBS (stimulated migration) were used in additional wells. AFSC were allowed to migrate for 24 hours. Membranes were then stained with crystal violet, rinsed in distilled water, eluted from the membrane using 0.1 M HCl and read at 600 nm in a spectrophotometer.

### In vitro AFSC Rescue

To determine the extent of the hypothesized direct interaction between AFSC and AECII during acute inflammation we devised an assay system, which employed *in vivo* bleomycin lung injury, followed by *in vitro* murine AFSC rescue. We isolated AECII from control or bleomycin-injured mice, three days post-intratracheal instillation of 1.5 U/kg bleomycin according to our previously published protocol [57]. Briefly, AECII from lavaged lungs were isolated by dispase digestion followed by differential adherence on IgG plates. Isolated AECII were plated in 6 well tissue culture plates (BD Falcon, Franklin Lakes, NJ) coated with fibronectin (Sigma-Aldrich, St. Louis, MO) at a density of 5×10^5^ cells/well. Cells were allowed to attach overnight. Once attached, 5×10^4^ murine AFSC were added to experimental wells and allowed to remain in culture with AECII for and additional 24 hours. At the end of the 24 hour period CCL2 levels in conditioned media were determined via ELISA (Invitrogen, Camarillo, CA) then conditioned media was assayed for the ability to induce collagen synthesis according to our previously described *in vitro* soluble collagen assay.

### In Vitro MMP-2 Inhibition

AECII from non-injured lavaged lungs were isolated by dispase digestion followed by differential adherence on IgG plates as previously described [57]. Isolated AECII were plated in 6 well tissue culture plates (BD Falcon, Franklin Lakes, NJ) coated with fibronectin (Sigma-Aldrich, St. Louis, MO) at a density of 5×10^5^ cells/well. Cells were allowed to attach overnight, before being injured with 100-mU/ml bleomycin. Two hours post-bleomycin injury 5×10^4^ murine AFSC with and without an MMP-2 specific inhibitor (MMPi), *cis-*9-Octadecenoyl-N-hydroxylamide, Oleoyl-N-hydroxylamide, [10 mM] in ethanol (EMB Biosciences, San Diego, CA) were added to experimental wells and allowed to remain in culture with AECII for and additional 24 hours. At the end of the 24-hour period conditioned media was collected and CCL2 levels in conditioned media was determined via ELISA (Invitrogen, Camarillo, CA). RNA from freshly cultured cells was extracted using the Qiagen RNeasy kit (Qiagen, Valencia, CA) according to the manufacturer’s instructions. Quantitative PCR for, 18S and MMP-2 was performed using a Roche Light Cycler 480. Each qPCR analysis was performed in triplicate.

### Data Presentation and Statistical Analysis

Data are expressed as mean ± SEM unless otherwise stated. Comparisons between two groups were determined using a two-tailed Student’s t-test. For multiple comparisons, one-factor analysis of variance was used, followed by the appropriate ad-hoc test as dictated by the normality and distribution of the data. All statistical analyses were performed using SigmaPlot 12 (Systat Software Inc., San Jose, CA). P values less than or equal to 0.05 were considered significant and expressed as *p<0.05; **p<0.001.

## Results

### AFSC Treatment Inhibits Fibrotic Parenchymal Destruction 28 Days Post-bleomycin Injury In vivo

**Figure 1:**
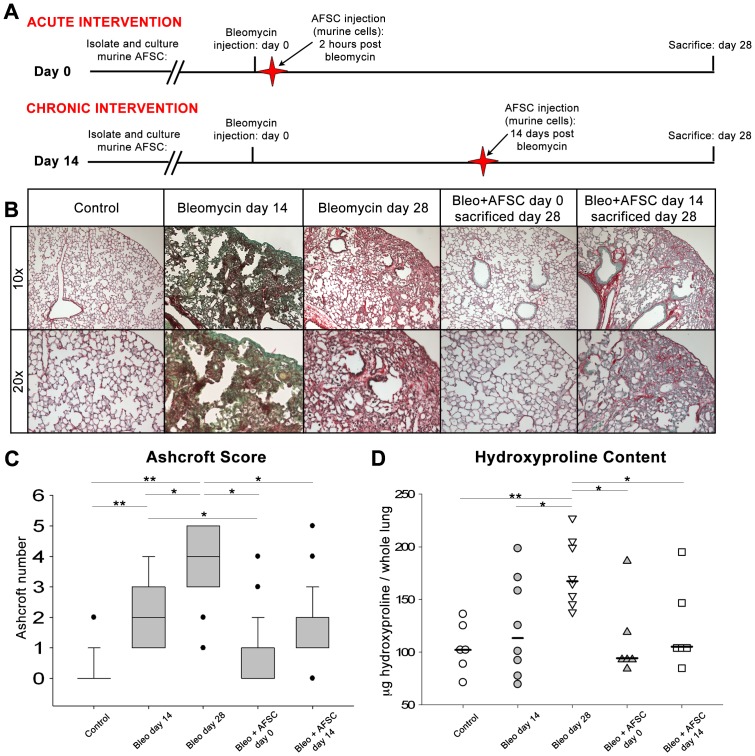
IV administration of murine AFSC inhibits fibrotic alveolar and parenchymal remodeling when injected during either acute or chronic periods following bleomycin induced lung injury. (A) IV AFSC injection was administered during either the acute period, 2 hours post-bleomycin injury, or during the chronic fibrotic remodeling period, 14 days post-bleomycin injury. Lungs were studied at day 28 post-bleomycin injury to visualize the full extent of fibrotic remodeling. Control animals: n = 6, day 14 and day 28 bleomycin injured: n = 8 per cohort, animals treated with murine AFSC two hours post bleomycin injury: n = 6, animals treated with AFSC 14 days post bleomycin injury: n = 6. (B) Histological analysis of adult mouse lung tissue embedded in paraffin, stained with Sirius Red/FCF Green, for collagen visualization examined at 10X and 20X. All collagen types-red; non-collagenous tissue-green/blue. (C) Ashcroft scoring of histological sections from bleomycin-injured mice. Distributions are presented as box plots with lines at the lower quartile, median and upper quartile, whiskers are representative of the minimum and maximums excluding outliers, dots are representative of outliers. (D) The measurement of total collagen content, as quantified by the hydroxyproline assay, was used to determine the amount of collagen present within the total lungs of the experimental cohorts. Distributions are presented as dot plots with lines indicating median values.

Histological specimens from control animals, bleomycin injured animals and animals receiving murine AFSC treatment IV during the acute (day 0) or chronic (day 14) phases of parenchymal destruction associated with bleomycin injury were stained with Sirius Red/FCF Green (Figure 1, A). Analyses showed no fibrotic lesions or alveolar destruction in control animals, while animals injured with bleomycin and sacrificed at day 14 post bleomycin injury displayed increased collagen deposition (red fibers) coupled with increased cellular infiltrate (green) and alveolar destruction resulting in the enlargement of airspaces. At 28 days post bleomycin injury, cohorts again exhibited alveolar destruction and increased collagen deposition (red fibers); however, cellular infiltrate present in day 14 sections was not observed (Figure 1, B). Animals treated with murine AFSC at day 0 and sacrificed 28 days later showed minimal fibrotic changes, limited to minor alveolar septal thickening and marginal alveolar destruction. Mice treated with murine AFSC at day 14 post bleomycin injury and sacrificed 28 days post injury, showed some collagen deposition similar to that observed in bleomycin injured cohorts harvested at day 14. Additionally, in cohorts treated with AFSC at day 14, alveolar destruction remained, however cellular infiltrate mostly observed occurring in distal subpleural regions of the lung (green), did not appear as prominent and was observed in non-AFSC treated cohorts.

The Ashcroft score for histological sections from control animals measured a median of 0 while bleomycin-injured lungs harvested 14 days post bleomycin-injury measured a median of 2 and bleomycin-injured lungs harvested 28 days post bleomycin-injury measured a median of 4 (p<0.001) [55]. In contrast, development of fibrosis in mice that received murine AFSC either at day 0 or day 14 was significantly diminished when compared to bleomycin injured cohorts, generating median Ashcroft scores of 1 and 2 respectively (p<0.05) (Figure 1, C). Furthermore, bleomycin-injured mice sacrificed 28 days post injury demonstrated a significant increase in measurable hydroxyproline content when compared to controls (p<0.001) or bleomycin injured mice sacrificed 14 days post injury (p<0.05). Mice treated with murine AFSC showed a significant reduction in lung hydroxyproline content when compared to bleomycin-injured cohorts sacrificed at day 28, whether AFSC were administered at day 0 (p<0.05) or at day 14 (p<0.05) (Figure 1, D). Sham injured control animals injected with murine AFSC at either day 0 or day 14 did not develop fibrotic lesions or display changes in hydroxyproline content (data not shown). These data demonstrate that AFSC treatment during either the initiating inflammatory events (2 hours post bleomycin injury) or the inception of fibrotic remodeling (14 days post bleomycin injury) significantly prevented the progression of further fibrotic remodeling.

### AFSC Treatment Inhibits Loss of Pulmonary Function Associated with the Development of Pulmonary Fibrosis 28 Days Post-bleomycin Injury In vivo

**Figure 2:**
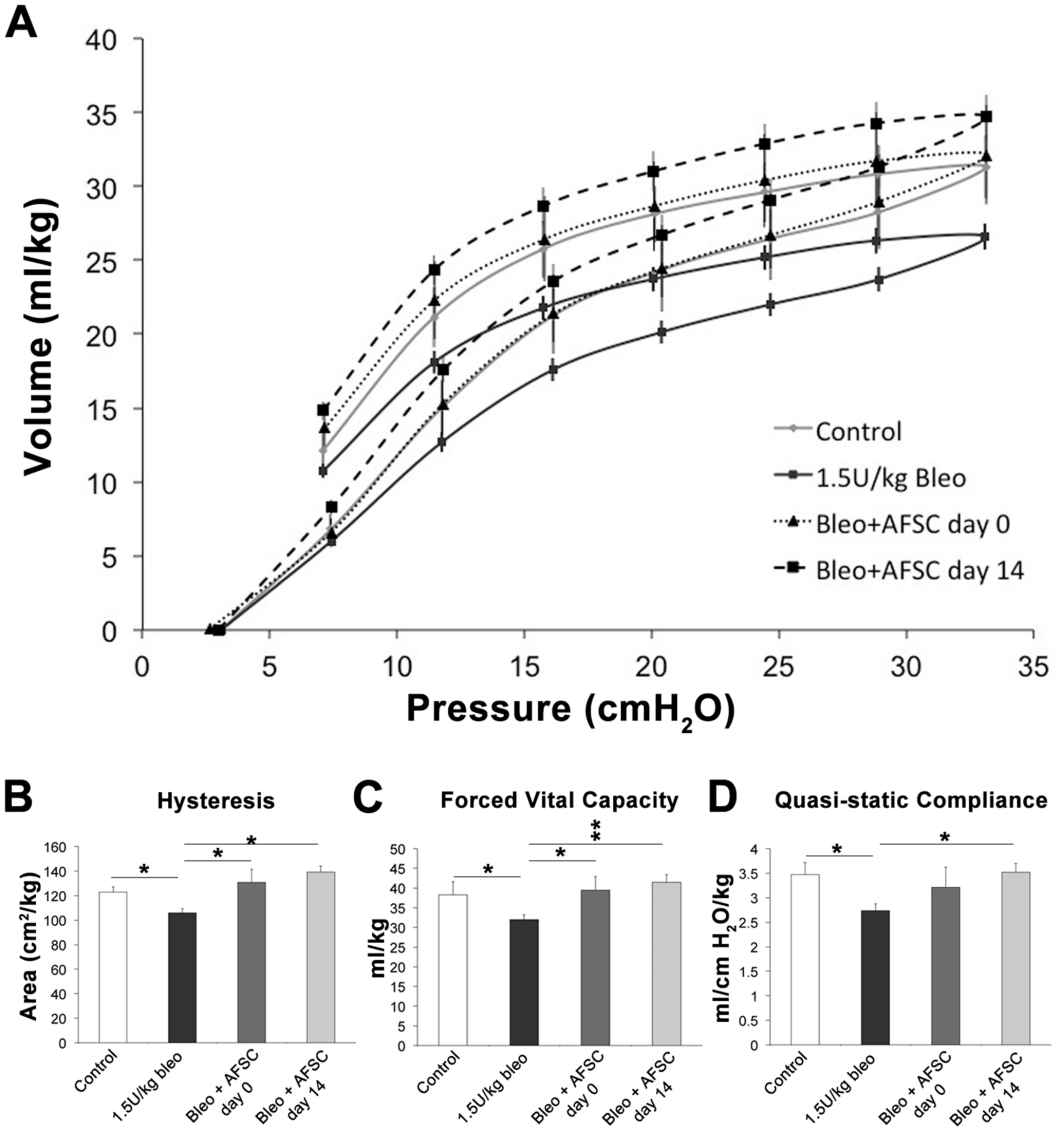
IV administration of AFSC attenuates loss of pulmonary function when injected during either acute or chronic periods following bleomycin induced lung injury. (A) Pressure-volume loops, describing the mechanical behavior of the lungs and chest wall during inflation and deflation (B) Area of hysteresis as calculated via the Salazar-Knowles equation. (C) Forced vital capacity. (D) Quasi-static compliance. Control animals: n = 5, bleomycin injured: n = 9, animals treated with murine AFSC two hours post bleomycin injury: n = 6, animals treated with murine AFSC 14 days post bleomycin injury: n = 6.

Pressure-volume (PV) loops describe the mechanical behavior of the lungs and chest wall during inflation and deflation. A shift of the PV-loop downwards along the volume axis occurs due to the development of fibrotic disease, indicating that more pressure is required to inflate the lungs to a given volume [56]. Following bleomycin injury, we indeed recorded a significant downward shift of the PV-loop along the volume axis as compared to control animals. Animals given murine AFSC at day 0 post-bleomycin injury displayed a P–V loop at day 28 nearly identical to control animals. Mice treated with murine AFSC at day 14 post-bleomycin injury showed an upward shift of the PV-loop along the volume axis as compared to control and bleomycin-injured animals. This upward shift indicates that less pressure was required to inflate the lungs to a given volume and could be attributable to the enlarged alveolar air-space size observed in both bleomycin-injured cohorts sacrificed at 14 days and “day 14” treated mice in Figure 1, B. (Figure 2, A), where the enlargement of airspaces and destruction of alveolar walls would contribute to the loss of elastic recoil, making the lungs easy to distend with a small change in pressure, as reflected in the pressure volume loop.

We applied the Salazar-Knowles equation to the PV-loop data to quantify hysteresis (the area contained within the pressure-volume loop) (Figure 2, B) [56]. When compared to control animals, bleomycin-injured mice showed a decrease in hysteresis (p<0.05). Animals that received murine AFSC treatment at either day 0 or day 14 showed an increase in hysteresis (p<0.05) when compared to bleomycin-injured mice. Following bleomycin injury, forced vital capacity routinely decreased (p<0.05), but was improved in cohorts treated with murine AFSC at day 0 (p<0.05) and day 14 (p<0.001) (Figure 2, C). Quasi-static compliance, which measures the elastic recoil pressure of the lungs at a given volume, decreased following bleomycin injury (p<0.05), but improved in both day 0 and day 14 (p<0.05) AFSC treated cohorts (Figure 2, D). Taken together, these results demonstrate that following AFSC treatment, at either of the two key events in bleomycin induced lung fibrosis, further loss of pulmonary function is impeded supporting our hypothesis that AFSC inhibit the progression of parenchymal remodeling associated with the development of fibrosis.

### AFSC Modulate Acute Inflammatory Cytokine Expression in BAL and Lung Tissue In vivo

**Figure 3:**
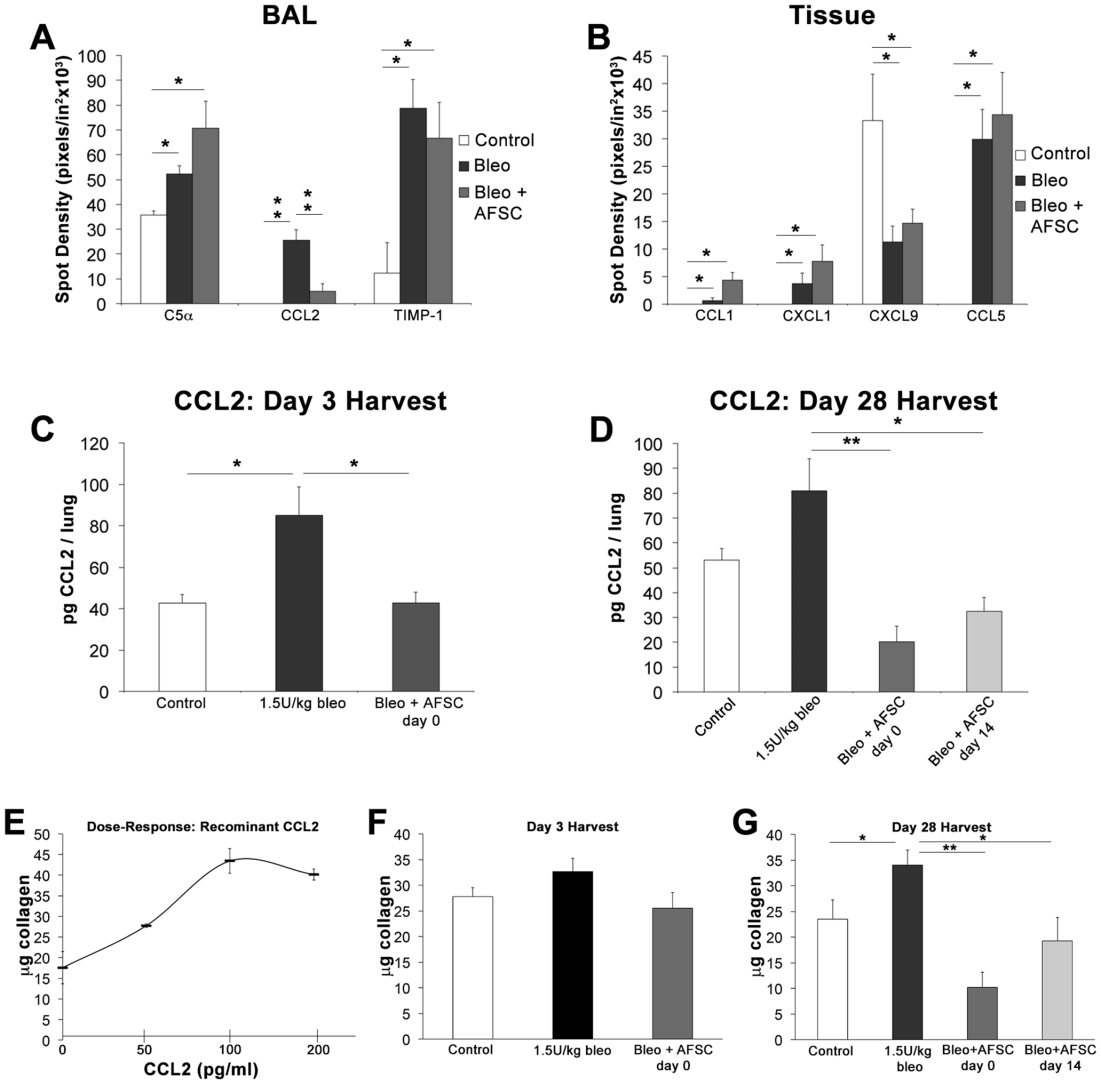
IV AFSC treatment modulates the acute inflammatory cytokine milieu in both BAL and tissue following bleomycin induced lung injury. Samples from BAL extracts (A) and whole lung homogenates (B) were analyzed via protein array to determine their acute inflammatory profiles. Cohorts included control (n = 6), bleomycin injured (n = 6) and bleomycin injured receiving AFSC treatment 2 hours post injury (n = 6). (C) CCL2 concentration in BAL quantified by ELISA during acute inflammation, 3 days post-bleomycin injury. (D) CCL2 concentration in BAL quantified by ELISA during the chronic injury period, 28 days post-bleomycin injury. (E) *In vitro* assay to determine the direct effect of varying concentrations of recombinant CCL2 on 3T3 fibroblast collagen synthesis. (F) Collagen synthesis induced in 3T3 fibroblasts following exposure to *in vivo* acute BAL samples. (G) Collagen synthesis induced in 3T3 fibroblasts following exposure to *in vivo* chronic BAL samples.

To test our hypothesis that AFSC exert immunomodulatory effects in response to bleomycin injury, we used proteomic arrays to examine BAL and lung tissue cytokine profiles 3 days post-bleomycin injury. Control, bleomycin-injured and bleomycin-injured mice that received murine AFSC treatment at day 0 were compared. BAL cytokine profiles demonstrated significant changes in C5α (p<0.05), CCL2 (p<0.001) and TIMP-1 (p<0.05) levels following bleomycin injury and AFSC treatment (Figure 3, A). Analysis of whole lung tissue homogenates (Figure 3, B) showed increases in CCL1, CXCL1 and CCL5 (p<0.05) and a decrease in CXCL9 (p<0.05) following both bleomycin injury and bleomycin injury with day 0 murine AFSC treatment.

Other cytokine modulations in BAL and lung tissue were detected, but were found not to be statistically significant (Figure 1S in the data supplement). Furthermore, the cellular component of BAL fluid, which was comprised mainly of macrophages, lymphocytes and neutrophils was analyzed to determine the effect of cytokine modulation on inflammatory cell populations (Figure 2S in the data supplement). Inflammatory cell recruitment to the lung, a key characteristic of the fibrotic and bleomycin injured lung [58]–[60], decreased overall following bleomycin injury, and increased moderately following murine AFSC treatment (Figure 2S, A). When individual cell populations were examined, cell populations, which express CCR2 and are thus responsive to CCL2 such as macrophages and lymphocytes increased after bleomycin injury and decreased following murine AFSC treatment (Figure 2S, B–C). Finally, neutrophils rose following murine AFSC treatment, which is consistent with complement activation C5α observed in murine AFSC treated cohorts (Figure 2S, D).

CCL2 concentrations in BAL from animals 3 days post-bleomycin injury were further quantified via ELISA. BAL collected from control mice exhibited CCL2 levels of 42.67±4.01 pg/lung, while CCL2 levels in BAL from bleomycin-injured animals increased 2-fold to 84.97±13.87 pg/lung (p<0.05). Animals that received murine AFSC at day 0 demonstrated a decrease in CCL2 levels to 42.78±5.10 pg/lung (p<0.05) (Figure 3, C). BAL collected from bleomycin-injured animals 28 days post-injury demonstrated an increase in CCL2 when compared to control animals (80.89±13.07 pg/lung versus 53.15±4.45 pg/lung), in contrast with animals that received murine AFSC at either day 0 or day 14, which showed significantly decreased levels of CCL2 at the 28 day post-injury time point (20.18±6.23 (p<0.001) and 32.48±5.49 pg/lung (p<0.05), respectively) (Figure 3, D).

CCL2 has been previously shown to promote collagen synthesis in fibroblasts though induction of the TGF-β pathway [14]. To determine what if any impact increased CCL2 concentrations found in BAL post-bleomycin injury could have on collagen synthesis by fibroblasts, 3T3 fibroblasts were exposed to increasing concentrations of recombinant CCL2 in culture. This resulted in a 2.5-fold increase in collagen synthesis when cells were exposed to 100 pg/ml CCL2, similar to CCL2 levels detected in bleomycin-injured murine BAL (Figure 3, E). BAL samples analyzed via ELISA in Figure 3 C–D were then used in this same collagen assay. BAL from mice 3 days post-bleomycin injury elicited a moderate but noticeable increase in collagen synthesis by 3T3 cells as compared to control BAL. Decreased levels of collagen were synthesized by 3T3 cells exposed to BAL from AFSC treated mice (Figure 3, F). BAL from animals 28 days post-bleomycin injury induced a significant increase in collagen synthesis when compared to control animals (p<0.05). Treatment of bleomycin-injured mice with AFSC at days 0 or 14 post-injury resulted in production of BAL that induced significantly less collagen synthesis when compared to BAL from bleomycin-injured mice, with 3.34-fold (p<0.001) and 1.77-fold (p<0.05) reductions in 3T3 collagen synthesis respectively (Figure 3, G). Increases in cell number following exposure to CCL2 or BAL samples were not statistically significant at the conclusion of the assay (data not shown), thus indicating that increases in collagen measured were due to increased collagen synthesis and not increases in cell number.

These data demonstrate that AFSC are immunomodulatory in the bleomycin lung injury model, and that AFSC have the capacity to modulate key pro-inflammatory/pro-fibrotic cytokines, most notably CCL2. The link to CCL2 mediated processes following bleomycin injury and AFSC rescue is further supported by the recruitment of CCL2 responsive immune cell populations into the BAL with and without AFSC treatment. Furthermore, these data show the direct impact of recombinant CCL2 alone or BAL containing varying levels of CCL2 (associated with AFSC treatment), on collagen synthesis by fibroblasts *in vitro*.

### AFSC Modulate CCL2 through MMP-2 Mediated Proteolytic Cleavage

**Figure 4:**
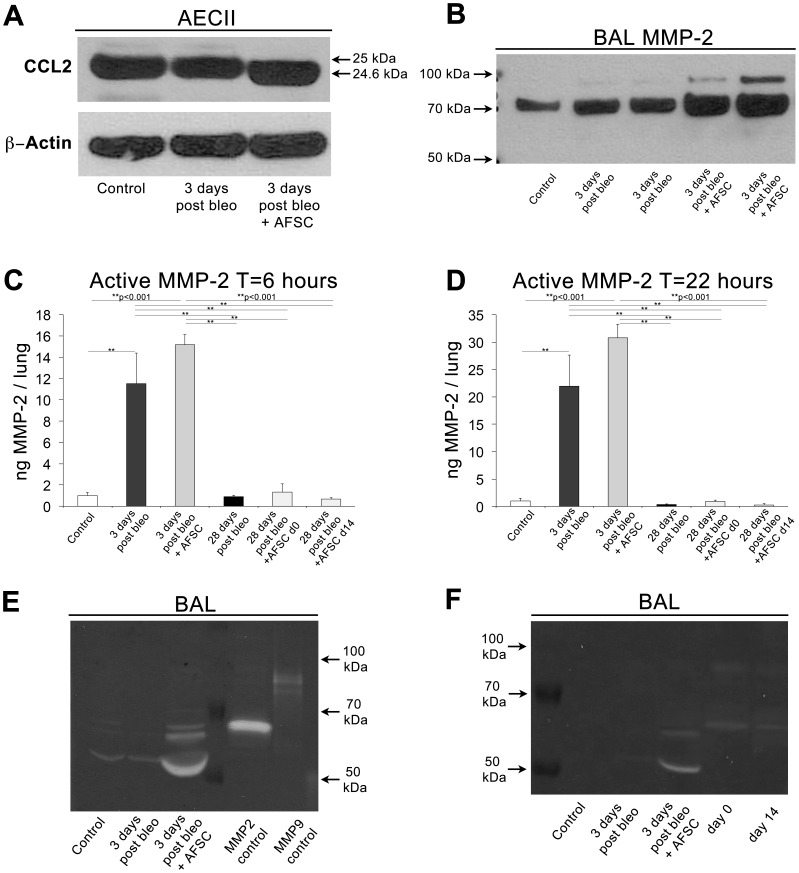
AFSC modulate AECII secreted CCL2 in BAL through proteolytic cleavage by transient MMP2 expression. (A) Representative Bis-Tris SDS-PAGE analysis of AECII fractions from control (n = 6), bleomycin-injured (n = 6) and bleomycin-injured with AFSC treatment at 2 hours post-bleomycin (n = 6), harvested at day 3, demonstrated a subtle 0.4 KDa shift of CCL2 to a putative inhibitory form. (B) Representative Bis-Tris SDS-PAGE analysis of BAL fluid containing 20 µg of total protein from control, bleomycin-injured and bleomycin-injured with AFSC treatment at 2 hours post-bleomycin, harvested at day 3 demonstrates increased MMP-2 expression in AFSC treated cohorts. (C–D) Measurement of endogenous active MMP-2 in BAL fluid at 6 and 22 hours of incubation demonstrating increased MMP-2 activity acutely following AFSC treatment, which is diminished 28 days post-bleomycin injury. (E) Representative gelatin zymography of BAL fluid containing 20 µg of total protein from control, bleomycin-injured and bleomycin-injured with AFSC treatment at 2 hours post-bleomycin, harvested at day 3 (F) Representative gelatin zymography of BAL fluid containing10 µg of total protein control, bleomycin-injured and bleomycin-injured with AFSC treatment harvested at day three as compared to BAL fractions from animals harvested at 28 days post-bleomycin injury (receiving AFSC at either day 0 or day 14 post-bleomycin injury), demonstrates transient nature of MMP2 increase.

To investigate a potential mechanism of CCL2 regulation in BAL following AFSC treatment, we performed Western blot analysis of CCL2 in AECII cellular fractions 3 days post-bleomycin injury (immunomodulatory time point of interest), using a gradient gel with high resolving capacity. Since we previously demonstrated that AFSC treatment most significantly attenuates CCL2 secreted into the BAL following bleomycin injury (Figure 3, A–D), Western blots of cell fractions were not used to measure changes in levels of CCL2 secretion, but instead as an indicator of the presence and type of CCL2. In controls, as well as following bleomycin injury, mouse-specific CCL2 was present as a band of the expected molecular weight, 25 kDa (Figure 4, A). However, following murine AFSC treatment, a subtle shift downward in the CCL2 band was visualized, indicating the cleavage of a 0.4 kDa peptide. This shortened, cleaved form of CCL2 has been previously reported to function as a CCR2 receptor antagonist, thereby rendering the cleaved form of CCL2 found here a putative CCR2 receptor antagonist in BAL [50]–[52].

CCL2 cleavage has previously been shown to occur in the presence of MMP-2 [50]. To confirm the hypothesis that the truncated form of CCL2 could be due to proteolytic cleavage, Western blots of BAL fluid, with the cellular fraction removed, demonstrated increased MMP-2 levels in bleomycin injured+AFSC treated cohorts (Figure 4, B). To determine the levels of endogenous active MMP-2 in BAL samples, we performed an MMP-2 activity assay and measured at 6 and 22 hours of incubation (Figure 4, C–D). Three days post bleomycin injury; MMP-2 was significantly increased when compared to controls (p<0.001). Also at the 3-day time point, AFSC treated cohorts exhibited marked increases in MMP-2 activity when compared to bleomycin-injured cohorts. MMP-2 activity significantly decreased at 28 days post bleomycin injury (p<0.001) and in day 0 and day 14 AFSC treated cohorts measured 28 days post bleomycin injury (p<0.001). Finally, gelatin zymography of BAL harvested 3 days post-bleomycin injury plus murine AFSC day 0 treatment showed a significant increase in MMP-2 activity (Figure 4, E). This effect was transient, as seen in the MMP-2 activity assay, as elevated levels of MMP-2 did not persist to 28 days and this was true whether AFSC were given at 0 or 14 days post-bleomycin injury. (Figure 4, F). These data are significant, as CCL2 is a known target of MMP-2 proteolytic cleavage, with the CCL2 cleavage product forming the aforementioned putative receptor antagonist for CCR2 [50]. These data suggest a potential mechanism for CCL2 modulation: the proteolytic cleavage of CCL2, forming a receptor antagonist, which maintains its binding affinity for CCR2, yet does not induce a response upon binding. We hypothesize that the transient increased MMP-2 secretion into BAL, detected after AFSC treatment, contributes to proteolytic cleavage of CCL2, thereby regulating its activity.

### AFSC Chemotactically Respond to Increased CCL2 Gradients

**Figure 5:**
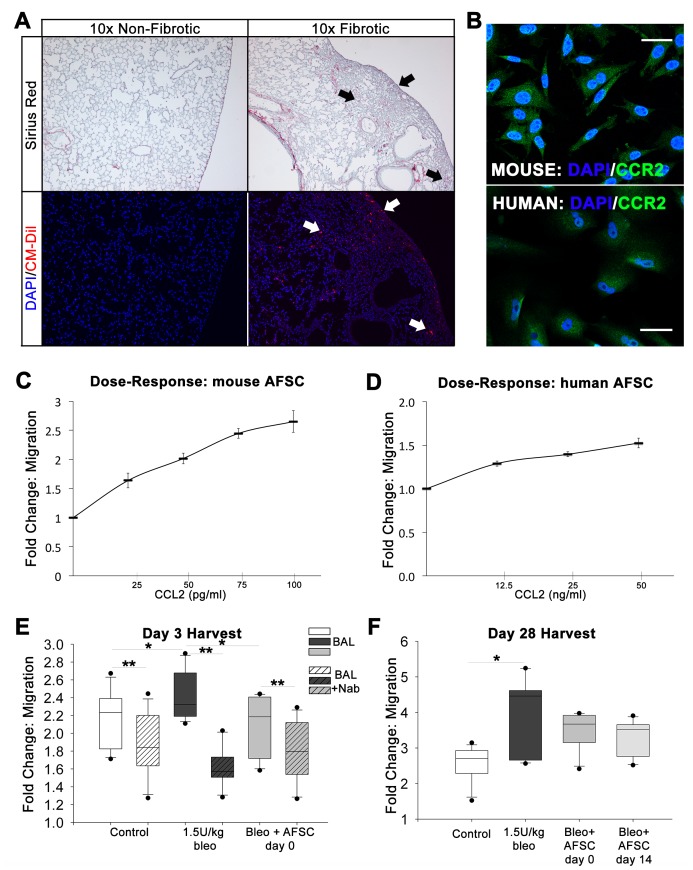
AFSC are retained within fibrotic lesions and migrate toward increased CCL2 concentrations. (A) Sections from lungs injured with bleomycin and injected with CM-Dil labeled AFSC 14 days post bleomycin injury, stained with Sirius Red/FCF Green and DAPI and visualized at 10× show increased retention of AFSC within fibrotic lesions (arrows). (B) Cultured human and murine AFSC express CCR2, the cognate receptor for CCL2, visualized by immunofluorescence. Scale bar = 50 µm. (C) Murine AFSC migrate toward a recombinant CCL2 gradient. (D) Migration elicited by CCL2 in human AFSC toward recombinant CCL2. (E) AFSC migration toward BAL harvested at day 3 from control, bleomycin-injured versus bleomycin-injured with AFSC treatment at day 0, assayed for the ability to chemoattract AFSC (gray boxes). Migration toward BAL with CCL2 neutralized using a neutralizing antibody (Nab) elicited a diminished migratory response in AFSC (hashed boxes). Distributions are presented as box plots with lines at the lower quartile, median and upper quartile, whiskers are representative of the minimum and maximums excluding outliers, dots are representative of outliers. (F) AFSC migration toward BAL samples harvested at the 28-day time point having either received no treatment, or treatment at days 0 or 14. Distributions are presented as box plots with lines at the lower quartile, median and upper quartile, whiskers are representative of the minimum and maximums excluding outliers, dots are representative of outliers.

Control mice injected with CM-Dil labeled murine AFSC did not exhibit fibrotic changes in lung tissue when analyzed using Sirius Red/Fast Green FCF and did not demonstrate retention of AFSC (data not shown). Mice injured with bleomycin and treated with murine AFSC at either day 0 or day 14 exhibited preferential AFSC retention within fibrotic regions of the lung when examined at day 28 (Figure 5, A). To rule out any contribution by AFSC to the development of fibrotic lesions, we analyzed α-SMA expression in murine AFSC in situ in bleomycin-injured and treated lung and did not observe co-localization of α-SMA expression with AFSC (data not shown).

It has been previously characterized that in both human IPF and murine bleomycin-induced lung injury increased CCL2 expression is noted within activated epithelium in fibrotic areas [7]. To determine if the presence of murine AFSC in fibrotic lesions in this study could be the result of chemotaxis toward areas of increased CCL2 expression, we analyzed murine AFSC prior to injection for CCR2 receptor expression. Immunofluorescent staining for CCR2 demonstrated that indeed murine AFSC express this receptor prior to injection. Additionally, staining for CCR2 on human AFSC indicated expression of the receptor as well (Figure 5, B).

Furthermore, to begin to investigate the translational potential of this hypothesized homing mechanism in humans, we assayed both human and mouse AFSC for chemotaxis *in vitro* towards increasing concentrations of recombinant mouse CCL2, which is a chemoattractant for both human and mouse cells that express CCR2 [61]. Murine AFSC migrated, in a dose dependent manner, toward increasing concentrations of CCL2 in culture (Figure 5, C). The greatest murine AFSC migration observed, toward a concentration of 100 pg/ml (similar to what is found in murine BAL following bleomycin injury), demonstrated a 2.24-fold increase when compared to AFSC not exposed to CCL2. Migration of human AFSC, assayed to determine the translational potential of this chemotactic response, demonstrated a moderate peak at 50 ng/ml, a CCL2 concentration similar to that reported in BAL of IPF patients (Figure 5, D) [62]. BAL samples previously analyzed via ELISA (Figure 3, C–D) were also tested for the ability to chemoattract murine AFSC. Murine AFSC were significantly more attracted to bleomycin-injured BAL than to BAL from control and day 0 AFSC-treated mice (p<0.05) (Figure 5, E) demonstrating the specificity of CCL2 as a potent chemoattractant for AFSC. Furthermore, upon CCL2 neutralization in BAL samples 3 days post-bleomycin injury using a CCL2 neutralizing antibody (Nab), migration toward control, bleomycin-injured, and bleomycin-injured plus AFSC day 0 treated samples all decreased significantly (p<0.001) (Figure 5, E). Finally, murine AFSC migration toward BAL samples harvested at day 28 post-bleomycin injury demonstrated a significant increase (p<0.05) in chemotaxis when compared to controls. Chemotaxis decreased in both day 0 and day 14 AFSC treated mouse BAL (Figure 5, F).

These experiments demonstrated that AFSC express CCR2, are preferentially retained within fibrotic lesions, and are not contributing to the deposition of collagen. Additionally AFSC can actively respond to the chemotactic gradient induced following bleomycin injury, specifically CCL2 within that injurious gradient, which once neutralized inhibits the chemotaxis of AFSC. Finally, we demonstrate the translational potential of human AFSC to respond to CCL2 gradients at concentrations found in human IPF patients.

### AFSC Co-cultured with Bleomycin Injured AECII Inhibit Increased CCL2 Expression In vitro

**Figure 6:**
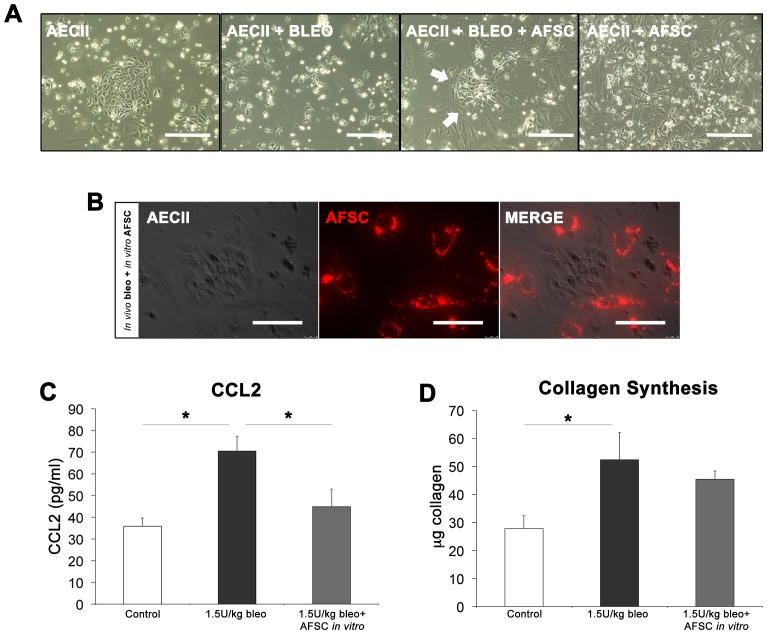
In vitro AFSC co-culture with *in vivo* injured AECII recapitulates *in vivo* CCL2 regulation. (A) Bright field microscopy of *in vivo* sham and bleomycin-injured AECII at 3 days post-injury cultured with and without AFSC. Scale bar = 200 µm. (B) Phase contrast microscopy of *in vivo* injured AECII co-cultured with CM-Dil stained (red) AFSC visualized via fluorescence microscopy. Scale bar = 100 µm. (C) *In vitro* levels of CCL2 in conditioned media as measured by ELISA. (D) Effect of conditioned media from *in vitro* AFSC co-culture experiments on induction of 3T3 collagen synthesis.

To examine the direct interaction between bleomycin-injured AECII, which secrete CCL2 [7], and AFSC during the acute inflammatory period, we injured mice with bleomycin or saline, harvested AECII 3 days post-injection, and then co-cultured AECII with murine AFSC. AECII harvested from saline injected animals grew in circular colonies on fibronectin-coated plates, while AECII from bleomycin injected animals grew sporadically and did not appear to attach well (Figure 6, A). Addition of AFSC to bleomycin-injured AECII in culture resulted in the AFSC surrounding bleomycin-injured AECII, which then formed colonies (arrows), while uninjured AECII co-cultured with AFSC did not demonstrate this same phenomenon. Further visualization with CM-Dil labeled AFSC and unlabeled AECII again demonstrated the phenomenon in which AFSC surround bleomycin injured AECII colonies (Figure 6, B).

In experiments on cultured AECII that paralleled our previous observations using BAL, elevated levels of secreted CCL2 in conditioned media as measured by ELISA were observed in bleomycin-injured AECII wells at 70.47±6.83 pg/ml compared to 35.85±1.68 pg/ml measured in control AECII wells (p<0.05). Conditioned media from wells containing AECII co-cultured with AFSC demonstrated a decrease in secreted CCL2 levels to 44.91±7.98 pg/ml (p<0.05) (Figure 6, C). Conditioned media from cultured cells isolated from bleomycin-injured lung induced a significant increase in collagen synthesis in 3T3 fibroblasts (p<0.05) as compared to control AECII conditioned media. This ability to stimulate collagen synthesis was reduced when injured AECII were co-cultured with AFSC (Figure 6, D). Although *in vivo* there are numerous cell types that contribute to the production of CCL2, these *in vitro* experiments demonstrate that the direct interaction between AFSC and injured AECII, the primary cell population responsible for CCL2 production in the fibrotic lung [7], results in CCL2 modulation similar to that seen in our *in vivo* modeling.

### Inhibition of MMP-2 *in vitro* Attenuates the Ability of AFSC to Reduce CCL2 Expression

**Figure 7:**
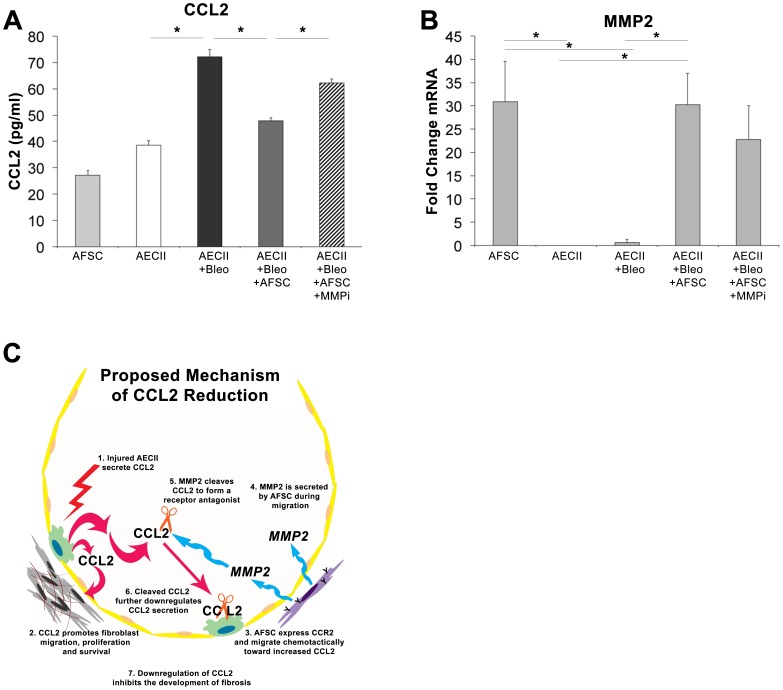
Inhibition of MMP2 *in vitro* inhibits the ability of AFSC to reduce CCL2 levels following AECII bleomycin injury. (A) CCL2 ELISA of conditioned media from *in vitro* injury of AECII with 100 mU/ml of bleomycin with AFSC co-culture with and without the addition of an MMP2 inhibitor. (B) qPCR analysis of cellular MMP2 expression from *in vitro* injury of AECII with 100 mU/ml of bleomycin with AFSC co-culture with and without the addition of an MMP2 inhibitor. (C) Proposed mechanism of AFSC mediated CCL2 modulation within the alveolus.

Finally, to test our hypothesis that MMP-2 plays a role in CCL2 regulation, we sought to determine if inhibition of MMP-2 using an MMP-2 specific inhibitor (Oleoyl-N-hydroxylamide) would restore increased secreted CCL2 levels observed in cultured AECII following bleomycin injury, we employed an assay in which AECII were injured with bleomycin *in vitro* and co-cultured with AFSC two hours post-injury. Control levels of CCL2 in AFSC and non-injured AECII conditioned media were measured at 27.18±1.78 pg/ml and 38.18±1.75 pg/ml, respectively (Figure 7, A). After AECII bleomycin injury *in vitro*, CCL2 levels in the media doubled to 72.13±2.68 pg/ml (p<0.05). Following co-culture with murine AFSC, CCL2 levels significantly decreased to 47.86±1.15 pg/ml. Finally, addition of the MMP-2 inhibitor to bleomycin-injured AECII co-cultured with AFSC resulted in a significant increase in CCL2 to 62.22±1.42 pg/ml (p<0.05). Furthermore, this increase in CCL2 was not significantly different from levels in wells that had experienced bleomycin injury alone. qPCR analysis of cellular MMP-2 (Figure 7, B) showed that mRNA levels of MMP-2 in AFSC were significantly higher than both uninjured and injured AECII prior to co-culture (p<0.05). Following *in vitro* AECII bleomycin injury and AFSC co-culture, MMP-2 mRNA remained elevated (p<0.05) yet showed a noticeable decrease, when compared to wells containing an MMP-2 inhibitor. These data demonstrate that CCL2 reduction following AFSC co-culture, is linked to increased MMP-2 expression by AFSC, and that inhibition of MMP-2 results in increased CCL2 levels that are statistically indistinguishable from levels expressed by AECII injured with bleomycin.

Based on the data in its entirety, we propose the following mechanism (Figure 7,C). Following AECII injury, CCL2 is secreted. CCL2 triggers fibroblast migration, proliferation and survival. AFSC express CCR2, the receptor for CCL2 and respond chemotactically to increased CCL2 gradients. MMP-2 is secreted during migration, a known mechanism utilized by highly motile cells [63]. Secreted MMP-2 cleaves CCL2 forming a receptor antagonist. Binding of the CCR2 antagonist either inhibits the recruitment of CCL2 secreting cell populations and/or triggers an autocrine effect as previously described [64] downregulating the further secretion of CCL2 by local cell populations, thus inhibiting the further development of CCL2 induced fibrosis. We suggest that the proposed mechanism of CCL2 reduction by AFSC is a temporal response, occurring as AFSC home to sites of injury. We surmise that AFSC home to fibrotic lesions during a period that CCL2 is increased and that subsequent secretion of MMP-2 by AFSC reduces CCL2 levels following localization within these lesions.

## Discussion

IPF is a disease that lacks both a cause and definitive treatment and is typically not diagnosed until the chronic stage in which fibrotic lesions have been established and patients present with diminished lung function [65]. The need for treatment strategies for IPF during clinically relevant diagnostic periods; targeted toward clinically relevant pro-fibrotic mediators is essential. Although the bleomycin injury model falls short as a perfect model for human idiopathic pulmonary fibrosis, certain aspects, which have been characterized in the literature and in the data presented, serve as useful facsimile for human IPF [46], [48], [49]. Careful consideration was taken in the selection of intervention periods and endpoints in this study to account for some of the known limitations of the bleomycin injury model, such as the spontaneous resolution of fibrotic lesions. Thus, we suggest that the data from our *in vivo* and *in vitro* models may be clinically and physiologically relevant to CCL2 dependent events characteristic of human IPF.

We have demonstrated that AFSC treatment inhibits changes in lung function associated with the development of bleomycin-induced fibrosis when administered during both acute and chronic fibrotic remodeling events. While the acute intervention (day 0) allowed us to investigate a novel mechanism of action of AFSC, the chronic intervention (day 14) provided data that are clinically relevant. Although lung function in day 14 treated cohorts could not fully be restored to normal levels, due to alveolar destruction caused by the development of fibrosis prior to treatment seen in untreated day 14 bleomycin injured histology, we demonstrated that following AFSC treatment lung function and destruction of the alveolar architecture did not progress to the fibrotic extent seen in untreated cohorts. In all cohorts, the inhibition of the development of fibrosis was demonstrated by a decrease in measured hydroxyproline content, measured Ashcroft score and the preservation of lung mechanics and pulmonary function.

Based upon clinical and experimental characterizations of cellular and molecular responses in human IPF, specifically exaggerated CCL2 expression [62], [66]–[68], we have presented data, which indicate that AFSC attenuate increased CCL2 in BAL in an experimental model of lung fibrosis. We demonstrated that transient and local MMP2 up regulation, by AFSC in BAL was associated with the proteolytic cleavage of CCL2, thus creating a localized CCL2/CCR2 antagonist within the injured alveolar milieu. We hypothesize that this antagonist downregulates further pro-fibrotic CCL2/CCR2 signaling, either through a direct interaction with CCR2 inhibiting a potential CCL2/CCR2 autocrine loop previously described in other CCR2 expressing cell populations [64] or through inhibition of the further recruitment of CCL2 secreting cell populations. Furthermore, it is possible that the introduction of AFSC, a CCR2 expressing population, into this pro-fibrotic system serves to “scavenge” increased CCL2 in BAL as hypothesized by Cardona et al. [69]. These hypotheses, could potentially explain the as of yet undetermined mechanism for decreased CCL2 levels, below those seen in control animals, in bleomycin injured cohorts receiving AFSC at day 0 or day 14 at the day 28 time point.

Our data indicate that CCL2 regulation by MMP-2-mediated proteolytic cleavage occurs acutely following AFSC treatment, yet elicits chronic CCL2 reduction *in vivo* in BAL. We surmise that this transient MMP-2 expression is sufficient to cleave excess CCL2 produced during the active disease state, yet transitory enough to avoid the parenchymal degradation typically associated with chronic up regulation of MMPs [70]. We supported this proposed mechanism through analyses of two, independent, *in vitro* AECII injury models in which significant, secreted CCL2 expression was attenuated following AFSC co-culture. Finally, it is important to note that in all experiments that utilized AFSC, CCL2 secretion was not completely abrogated. The maintenance of a minimal level of CCL2 following AFSC treatment, may be critical for the protection of the homeostatic arm of the CCL2/CCR2 signaling pathway which not only participates in immune cell recruitment to areas of infection but also osteoclast differentiation and metabolic regulation [61], [71], [72]. Another potential impact of these data lies in the ability of AFSC to not only target their salutary therapeutic properties during clinically relevant intervention periods, but to home to the diseased region of the lung, foregoing non-diseased regions, as seen in our *in vitro* migration assays and *in vivo* histology that shows AFSC chemotaxis toward CCL2 and retention within fibrotic regions, respectively.

AFSC therapy, unlike previously published MSC based therapy, has yet to show deleterious secondary effects such as tumorogenesis or expression of fibrotic phenotypes in experimental models of chronic fibrotic injury [42], [44], [45], [73]. Furthermore, unlike specific CCL2 inhibitors, these studies coupled with our previously published findings demonstrate the plasticity of the mechanisms of action of AFSC, which are dependent on the type and location of injury. Although this study focuses on the effect of AFSC specifically on CCL2, it is likely that additional, undescribed mechanisms are involved in the antifibrotic effects of AFSC, such as those investigated in other models of fibrotic injury coupled with AFSC treatment [45]. We suggest that this makes AFSC perhaps more translationally applicable in the context of disease than many single agent systemic drug therapies. Our findings demonstrate the efficacy of AFSC within the bleomycin injury model and provide data, which suggest a novel mechanistic role for AFSC regulation of CCL2 resulting in the inhibition of parenchymal remodeling and the development of pulmonary fibrosis. These data provide insight into the potential tractability of targeting the CCL2/CCR2 pathway in fibrotic lung diseases via a novel AFSC cell-based therapy, and provides a treatment strategy that we think deserves further evaluation.

## Supporting Information

Figure S1 **AFSC modulation of the acute inflammatory cytokine milieu in both BAL and tissue following bleomycin induced lung injury.** (A) Table of all cytokine modulations detected in BAL. (B) Graph of samples from BAL extracts that were moderately, but not statistically significantly modulated. (C) Table containing all cytokine modulations detected in tissue homogenates. (D) Graph of samples from tissue homogenates that were moderately, but not statistically significantly modulated.
(TIF)

Figure S2 **AFSC modulation of the acute inflammatory cellular populations in BAL following bleomycin induced lung injury.** (A) Total cell count modulations detected in BAL. (B) Differential BAL macrophage analysis. (C) Differential BAL lymphocyte analysis. (D) Differential BAL neutrophil analysis. Distributions for B–D are presented as box plots with lines at the lower quartile, median and upper quartile, whiskers are representative of the minimum and maximums excluding outliers.
(TIF)
