# Supplementary figures and images for: Amniotic Fluid Stem Cells Inhibit the Progression of Bleomycin-Induced Pulmonary Fibrosis via CCL2 Modulation in Bronchoalveolar Lavage
Source: PLoS One. 2013 Aug 13;8(8):e71679. doi: 10.1371/journal.pone.0071679 (PMC3742516; doi:10.1371/journal.pone.0071679)

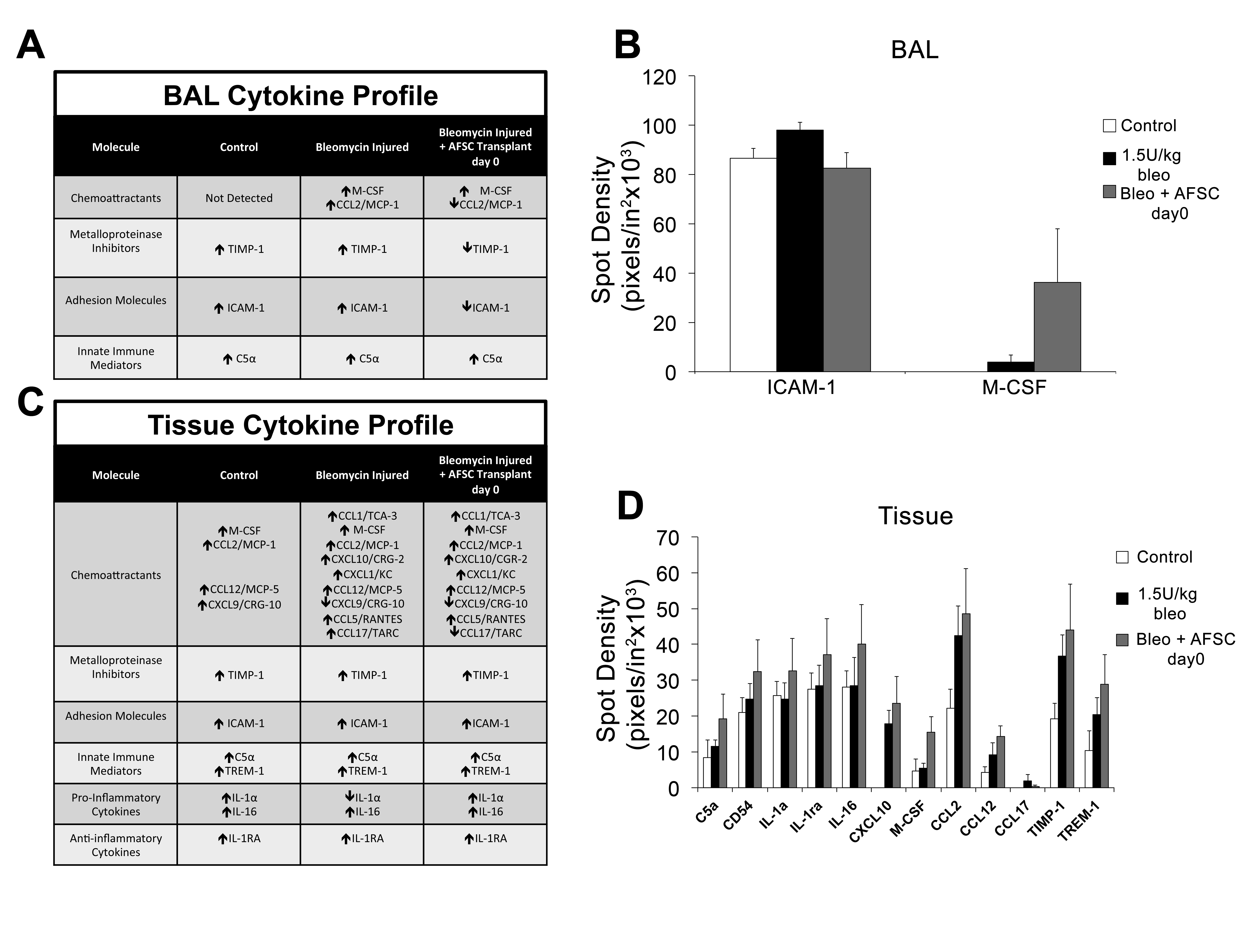

Supplement: Figure S1 — AFSC modulation of the acute inflammatory cytokine milieu in both BAL and tissue following bleomycin induced lung injury. (A) Table of all cytokine modulations detected in BAL. (B) Graph of samples from BAL extracts that were moderately, but not statistically significantly modulated. (C) Table containing all cytokine modulations detected in tissue homogenates. (D) Graph of samples from tissue homogenates that were moderately, but not statistically significantly modulated. (TIF) [file pone.0071679.s001.tif]

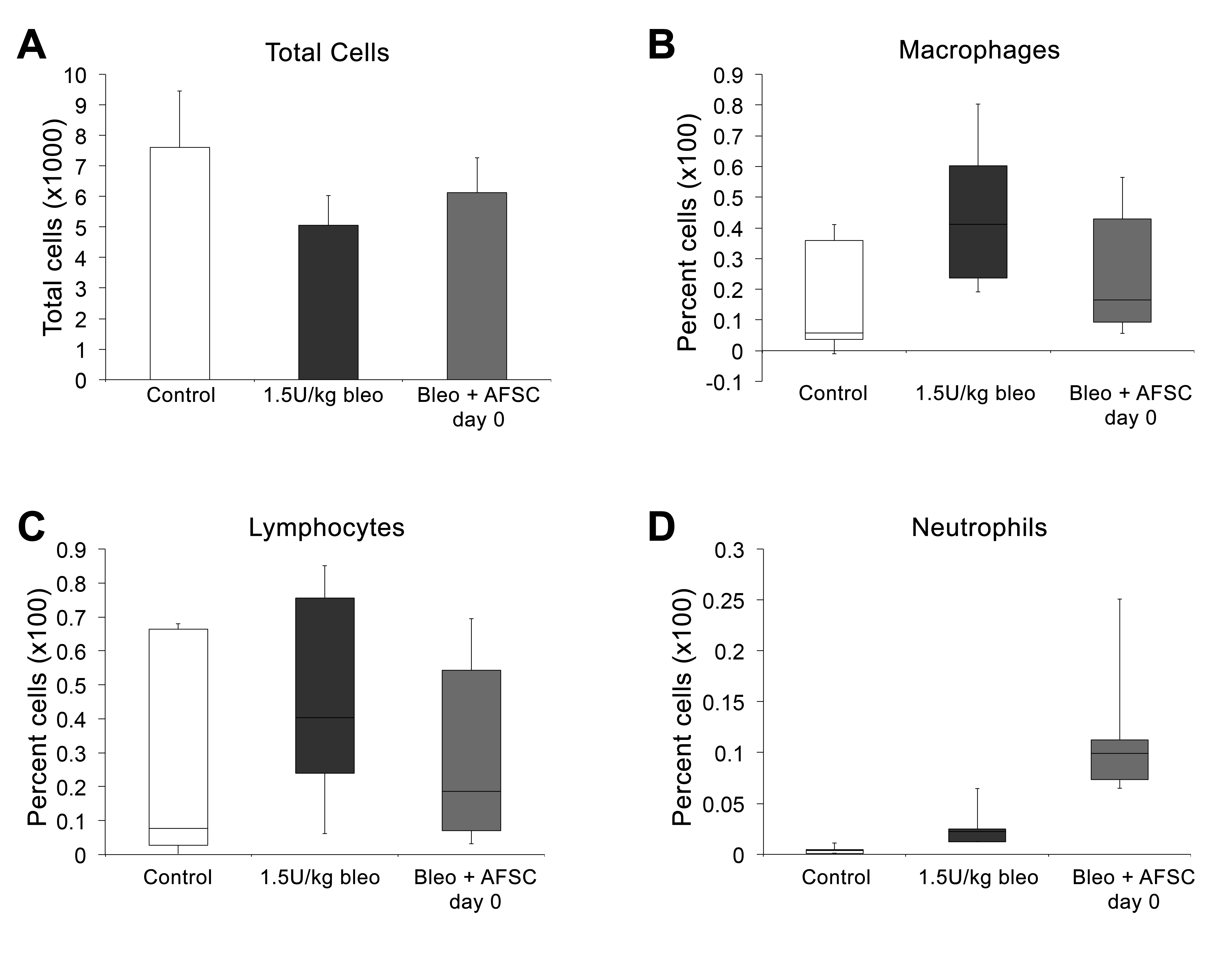

Supplement: Figure S2 — AFSC modulation of the acute inflammatory cellular populations in BAL following bleomycin induced lung injury. (A) Total cell count modulations detected in BAL. (B) Differential BAL macrophage analysis. (C) Differential BAL lymphocyte analysis. (D) Differential BAL neutrophil analysis. Distributions for B–D are presented as box plots with lines at the lower quartile, median and upper quartile, whiskers are representative of the minimum and maximums excluding outliers. (TIF) [file pone.0071679.s002.tif]
